# Catastrophic floods and antimicrobial resistance: Interconnected threats with wide-ranging impacts

**DOI:** 10.1016/j.onehlt.2024.100891

**Published:** 2024-09-10

**Authors:** João Pedro Rueda Furlan, Fábio Parra Sellera, Nilton Lincopan, Daniela Debone, Simone Georges El Khouri Miraglia, Ronan Adler Tavella

**Affiliations:** a Paulista School of Medicine, Federal University of São Paulo, São Paulo, Brazil; b ARIES, Antimicrobial Resistance Institute of São Paulo, São Paulo, Brazil; c Department of Internal Medicine, School of Veterinary Medicine and Animal Science, University of São Paulo, São Paulo, Brazil; d School of Veterinary Medicine, Metropolitan University of Santos, Santos, Brazil; e Department of Microbiology, Instituto de Ciências Biomédicas, University of São Paulo, São Paulo, Brazil; f Department of Clinical Analysis, Faculty of Pharmacy, University of São Paulo, São Paulo, Brazil; g Institute of Environmental, Chemical and Pharmaceutical Sciences, Federal University of São Paulo, Diadema, Brazil

**Keywords:** Climate change, Global warming, Multidrug-resistant microorganisms, Emerging pathogens, Public health

## Abstract

• Climate change and AMR combined worsen vulnerabilities, accelerating AMR spread.
• Floods can spread AMR-related pathogens, impacting health, agriculture, and ecosystems.
• Integrated strategies are needed to address climate change and AMR, enhancing sanitation.

Climate change and antimicrobial resistance (AMR) are among the ten threats to global health, according to the World Health Organization (WHO) [1]. These two crises, while distinct, are increasingly interconnected. Climate change alters ecosystems, agricultural patterns, and human migration, which can accelerate the spread of antimicrobial-resistant microorganisms. For instance, extreme weather events, such as floods, can disrupt healthcare services and sanitation systems, leading to increased use of antimicrobials in vulnerable populations and further fostering the development of AMR. This combination of threats is hazardous in fragile and vulnerable settings with weak primary healthcare infrastructures, especially in low- and middle-income countries, where climate-sensitive diseases and poor infection control mechanisms already strain health systems. Recently, the connection between climate change, biodiversity loss, and infectious diseases [2], and the One Health systems-thinking approaches to quantify anthropogenic-derived AMR [3] have been discussed. However, the linkage of these concerns has not been addressed, despite the possibility that catastrophic events related to climate change may speed up the transmission and evolution of AMR.

According to the Intergovernmental Panel on Climate Change, future climatic projections indicate an increased frequency of extreme precipitation events in Latin America, particularly in the South-Eastern region. When these events occur in non-resilient areas, they often result in catastrophic floods [4]. In urban settings, these floods expose people and infrastructure to contaminated waters as wastewater that harbors antimicrobial-resistant pathogens, posing a significant and silent threat to public health [5]. Properly, the dispersion of contaminated flood waters increases the likelihood of epidemic outbreaks, making disease control and treatment difficult. On the other hand, the implications of such exposure, intensified and further spread by flooding, reach far beyond urban areas, impacting rural, coastal, and natural sectors.

The agricultural sector can be severely affected, leading to broader economic and food security issues, whereas natural ecosystems are also disrupted, which can have long-term ecological consequences. Flood waters can contaminate soils and irrigation channels, affecting crop and livestock production. Accordingly, antimicrobial-resistant microorganisms, including WHO priority pathogens, can be introduced and spread into the food chain, further expanding the magnitude of the problem (Fig. 1). Moreover, antimicrobial use in agriculture, coupled with improper waste management, plays a significant role in the environmental dissemination of superbugs, exacerbating this issue [6].

**Fig. 1:**
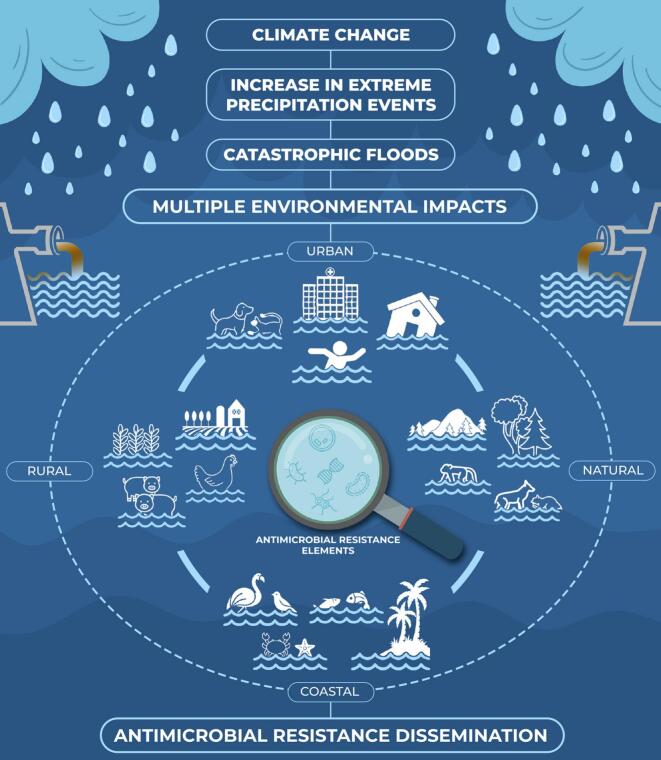
This infographic highlights the multiple environmental spread of AMR exacerbated by catastrophic floods. Precipitation events linked with global climate change are increasing floods in several countries, favoring the dissemination of AMR throughout urban, rural, natural, and coastal environments. These interconnected challenges pose wide-ranging impacts, exposing humans, animals, and their shared environments to underestimated consequences.

Natural ecosystems can also be disrupted, leading to long-term ecological consequences. It is presumed that exposure of wildlife to contaminated waters, soils, and sediments could increase the risk of colonization or infection by these pathogens. Coastal cities affected by such disasters might experience marine environmental contamination, including impaired beach water quality and contamination of marine fauna, which could compromise the fishing industry and local marine biodiversity. Migratory wild species visiting these areas could also be exposed, potentially contributing to the long-distance spread of AMR (Fig. 1). Hence, One Health surveillance studies aimed at monitoring microbial communities and superbugs in flood-affected areas are worth investigating.

Instances linking catastrophic floods to AMR have been observed in Asia, where surface and groundwater samples were analyzed after a major flood event in Chennai, India, in December 2015. The floods contaminated wells with sewage, leading to elevated bacterial counts and chemical ion levels beyond recommended limits. Worryingly, bacteria found in the groundwater exhibited resistance to medically important antimicrobials, highlighting the third-generation cephalosporin ceftriaxone [7]. Similar findings were observed in urban areas affected by hurricanes in North America. In August 2017, Hurricane Harvey, a Category 4 storm, inundated the Houston metropolitan area in the United States with rainfall equivalent to an entire year's worth, causing severe flooding in numerous wastewater treatment plants and damaging thousands of homes. Scientific reports have shown that AMR genes were more prevalent in samples collected shortly after the flooding compared to those taken much later [8,9].

Indeed, it was demonstrated that urban flooding events significantly alter the AMR profiles of soil-derived bacteria, potentially increasing the risk of exposure for up to three to five months post-hurricane; however, the prevalence of specific AMR genes decreased eighteen months after the event, suggesting a reduction in long-term exposure risk following the hurricane event [8]. In addition, sediments displaced by floodwaters from Hurricane Harvey showed a higher presence of potential pathogens after the flooding, both in residential areas and public parks [9]. These scenarios highlight the urgency of strengthening sanitation infrastructures and implementing effective water management measures to prevent environmental contamination and protect public health.

For instance, Brazil, the largest tropical country, has been facing significant challenges from natural disasters and the increasing prevalence of AMR at the human-animal-environment interface. Nevertheless, these issues have been mostly addressed independently. The recent floods in May 2024 in the Rio Grande do Sul, which persisted for 37 days and damaged more than 95 % of the cities in the state, covering an area of approximately 3.8 thousand km^2^_,_ demonstrate how natural disasters can amplify public health threats like AMR. The floodwaters not only disrupted economic activities in this region — home to the country's fourth-largest gross domestic product — but also likely facilitated the spread of AMR through contamination of water systems and agricultural lands. Accordingly, the need to integrate climate resilience [10] with AMR strategies should be discussed.

In summary, the global economic, social, ecological, and public health burden caused by floods is already presumed to be enormous. However, the impact could be even greater when considering the ramifications of AMR in the context of these climatic disasters. Addressing these interconnected challenges requires comprehensive and integrative approaches that consider the interplay between natural disasters and AMR, aiming to develop broad strategies to mitigate their combined impacts on public health, agriculture, and the environment. Tackling this issue aligns with the health-centered climate-resilient development of regions emphasized in the recent 2023 Latin America report of the Lancet Countdown on Health and Climate Change [11]. Therefore, the scientific community plays an important role in connecting public health authorities, political leaders, decision-makers, and citizens through a common and reliable language.

## Funding

The authors thank the 10.13039/501100001807São Paulo Research Foundation (grant nos. 23/16216-4, 23/04466-6, 24/02476-7, and 24/02579-0), the 10.13039/501100003593National Council for Scientific and Technological Development (grant nos. 308378/2021-0 and 314336/2021-4), and the 10.13039/501100002322Coordination for the Improvement of Higher Education Personnel (grant nos. 88887.463868/2019-00 and Finance Code 001) for fellowships.

## Ethical approval

Not required.

## CRediT authorship contribution statement

**João Pedro Rueda Furlan:** Writing – review & editing, Writing – original draft, Investigation, Formal analysis, Data curation, Conceptualization. **Fábio Parra Sellera:** Writing – review & editing, Writing – original draft, Investigation, Formal analysis, Data curation, Conceptualization. **Nilton Lincopan:** Writing – review & editing, Writing – original draft, Investigation, Formal analysis, Data curation, Conceptualization. **Daniela Debone:** Writing – review & editing, Writing – original draft, Investigation, Formal analysis, Data curation, Conceptualization. **Simone Georges El Khouri Miraglia:** Writing – review & editing, Writing – original draft, Investigation, Formal analysis, Data curation, Conceptualization. **Ronan Adler Tavella:** Writing – review & editing, Writing – original draft, Investigation, Formal analysis, Data curation, Conceptualization.

## Declaration of competing interest

The authors declare that they have no known competing financial interests or personal relationships that could have appeared to influence the work reported in this paper.

## Data Availability

No data was used for the research described in the article.
